# Commentary: The Boy Who Cried Wolf or Cassandra? A Consideration of the Correct Characterization of Critics of Neoliberal Reforms to the English NHS

**DOI:** 10.1177/27551938221148370

**Published:** 2023-01-04

**Authors:** David I. Benbow

**Affiliations:** 1 School of Law, 7315 University of Sheffield, Sheffield, UK

**Keywords:** English NHS, market incrementalism, ideology, privatization

## Abstract

Peter Roderick and Allyson Pollock's article, “Dismantling the National Health Service in England,” provides a history of the market incrementalism that has dominated UK government policy pertaining to the English National Health System (NHS), in recent decades. It also contains an analysis of the latest statute to reform the English NHS, namely the Health and Care Act 2022. It is often argued that the concerns—for example, about privatization—of those who critique neoliberal reforms to the English NHS are misplaced. I highlight that such neoliberal reforms have increased the proportion of the budget of the English NHS that is being diverted to private providers. Consequently, I aver that the term privatization accurately describes what has been occurring within the English NHS. I contend that the arguments of those who deny or downplay the privatization of the English NHS are indicative of some of the ideological strategies that the sociologist John B. Thompson identified. My commentary suggests that the concerns of critics of neoliberal reforms to the English NHS, such as Roderick and Pollock, are not misplaced and that more heed should be given to their analyses and warnings.

The founding principles of the National Health Service (NHS) in the United Kingdom were that it was to be: free, funded from general taxation, universal, and comprehensive. There is continued public support for such principles. For example, “the overwhelming majority of respondents” to the NatCen's British Social Attitudes Survey agreed that the founding principles of the NHS should “definitely” or “probably” apply in 2021.^1^ Such principles can be viewed as a moral economy.^2^ E.P. Thompson defined a moral economy as a popular consensus regarding legitimate and illegitimate practices based on a “traditional view of social norms and obligations.”^3^ Although successive UK governments have continued to valorize the NHS's founding principles, perennial underfunding and market reforms have eroded them within England. Health care is a devolved competence in Northern Ireland, Scotland, and Wales, hence policies differ in those parts of the United Kingdom. Peter Roderick and Allyson Pollock's article, “Dismantling the National Health Service in England,” provides a history of the market incrementalism that has dominated UK government policy pertaining to the English NHS in recent decades, together with an analysis of the latest statute to reform the English NHS, namely the Health and Care Act 2022.^4^

The recent market reforms to—and underfunding of—the English NHS (particularly from 2010 onward) have generated much opposition. Allyson Pollock has been one of the leading academic opponents of market incrementalism and has written powerful analyses of the gradual erosion of the founding principles of the NHS within England.^5^ I understand that Pollock first collaborated with Roderick to critique the bill that became the much-maligned Health and Social Care (HSC) Act 2012.^6^ Roderick and Pollock co-wrote the NHS (Reinstatement) Bill, which has been introduced, as a private members bill, into Parliament on a few occasions, but which has not proceeded to the statute book.^7^ The bill proposes to reverse the reforms that have marketized and privatized the English NHS.^7^ Pollock's critics have argued that her warnings of privatization are misplaced^8^ and that she is akin to the shepherd boy who cried wolf in Aesop's famous fable.^8,9^ I argue, within this commentary, that the Trojan priestess and prophetess, Cassandra, whose warnings of impending catastrophe were ignored, is a more apt analogy.^10^

Roderick and Pollock summarize the history of market incrementalism within the English NHS within their article.^4^ As they note, the first NHS quasi-market, which introduced the purchaser–provider split and was known as the internal market, was created via the National Health Service and Community Care Act 1990.^4^ Roderick and Pollock highlight how the market reforms continued with the Labour governments between 1997 and 2010, which gradually extended patient choices, ultimately to any willing provider (including the private sector) for some services.^4^ Labour also “scaled up” the Private Finance Initiative (PFI), paving “the way for what in effect became private sale-and-lease-back arrangements for new hospitals and services.”^4^ This has resulted in large amounts of money being extracted from the English NHS. For example, it is estimated that PFI profits for shareholders totaled almost £2 billion between 2010 and 2021.^11^ There is concern about potential abuse of market power as only 8 companies have equity stakes in 92% of PFI schemes within the English NHS.^12^ Additionally, Labour established foundation trusts (FTs) that were empowered to form joint ventures with private companies.^4^ The Conservative–Liberal Democrat coalition government's HSC Act 2012 created Clinical Commissioning Groups to commission secondary care services. As Roderick and Pollock note, the regulations passed pursuant to S.75 of that statute,^13^ “introduced virtually compulsory commercial tendering of contracts.”^4^ The HSC Act 2012 also permitted FTs to derive 49% of their income from private patients, which, as Roderick and Pollock lament, has diverted NHS staff and resources to private patients.^4^ The impact of austerity and the COVID-19 pandemic have also benefited the private sector, with many patients opting to go private rather than endure long waits for NHS treatment.^14,15^

Roderick and Pollock note that there were workarounds to the HSC Act 2012, in the years following its enactment, in a purported effort to achieve integrated care.^4^ This led to the creation of Integrated Care Boards (ICBs), which have been put on a statutory footing by the Health and Care Act 2022.^16^ In their thorough analysis, Roderick and Pollock identify many of the problematic aspects of the 2022 statute.^4^ For example, they identify similarities between ICBs and US health maintenance organizations, such as their funding, the populations covered, and the services provided.^4^ Health maintenance organizations were the first of many managed care organizations that have been adopted in the United States and elsewhere, the latest of which are the accountable care organizations created by Obamacare.^17^ Such managed care organizations in both the United States and in Latin American states have sought to exclude unprofitable patients.^18,19^ In England, Roderick and Pollock note that it is not clear whether patients will be able to choose ICBs or whether ICBs will be able to select patients.^4^ They also note that ICBs will have “core responsibility”^20^ for the group of people allocated to it, a concept that has not been explained, but which implies further erosion of the comprehensiveness of the English NHS.^4^ Roderick and Pollock highlight that ICBs will also be less accountable and less transparent than their predecessors, Clinical Commissioning Groups.^4^

The 2022 statute repeals the HSC Act S.75 and the controversial regulations passed pursuant to them.^21^ Roderick and Pollock believe that this “opens the way for cronyism.”^4^ Such cronyism was in evidence during the COVID-19 pandemic, when many contracts were awarded to private companies under special powers that circumvented normal tendering rules.^22^ Roderick and Pollock contend that power within the reformed English NHS will increasingly lie with provider collaboratives, including private companies, such as US health insurers and providers United Health (Optum) and Centene (Operose), which will have the responsibility for designing services and the discretion to determine how services are delivered.^4^ There has been concern about potential conflicts of interest if agents of provider collaboratives are able to sit on ICBs and/or their committees.^4^ Such concerns led to amendments to the statute that stipulate that ICB constitutions must prohibit appointments if they “could reasonably be regarded as undermining the independence of the health service because of the candidate's involvement with the private healthcare sector or otherwise.”^23^ In other analyses of the legislation, Mary Guy states that the changes to the procurement rules seem to represent a refocus rather than a removal of competition^24^ and Albert Sanchez-Graells speculates that they could generate more disputes and litigation.^25^ Ultimately, Roderick and Pollock aver that the reforms of the 2022 statute “make further development of a two-tier and mixed-funding system inevitable.”^4^ They lament that the result will be a health care system, within England, characterized by “high costs, inequality, and injustice.”^4^

This latest analysis of reforms to the English NHS by Roderick and Pollock may lead to further comparisons with the shepherd boy in Aesop's fable.^9^ However, as mentioned above, a more apt analogy can be made with another figure from Ancient Greek literature, namely, Cassandra, whose prophecies were not believed.^10^ As Roderick and Pollock highlight, “successive governments, think tanks and the mainstream media repeatedly” deny “that the NHS is being privatized.”^4^ The World Health Organization defined privatization as “a process in which nongovernmental actors become increasingly involved in the financing and/or provision of healthcare services.”^26^ This accurately describes what has been occurring within the English NHS. Policies that enable private companies to profit from public services covertly redistribute wealth to the affluent and powerful.^27^ John B. Thompson defined ideology as the way “in which meaning (or signification) serves to [establish and] sustain relations of domination.”^28^ Thompson identified various modes of ideology (such as legitimation, dissimulation, unification, fragmentation, and reification) and their strategies (e.g., narrativization is a strategy of the legitimation mode and naturalization is a strategy of the reification mode).^29^ The arguments of those who deny or downplay such privatization demonstrate such strategies.

For example, Mark Dayan and Helen Buckingham contended that those concerned with privatization, in relation to the bill that became the Health and Care Act 2022, are “missing the point.”^30^ Dayan and Buckingham narrativize the involvement of private companies as legitimate. For example, they state that “in reality, the NHS has paid private providers to deliver free care ever since it was founded in 1948.”^30^ This downplays the large increase in private sector involvement in the English NHS within the neoliberal era. They also naturalize this involvement, as though there could not be an alternative. Although Dayan and Buckingham acknowledge that the NHS paying private firms to provide services constitutes privatization, they aver that the ostensible stable level of private provision in the 6 years prior to 2021 belies accusations of increasing privatization.^30^ However, their reasoning is based on flawed data from the Department of Health and Social Care's annual reports and accounts, which, as David Rowland notes, exclude payments to local authorities and the voluntary sector and major items of expenditure on the private sector (such as the amount that NHS trusts purchase from the independent sector).^31^ Consequently, the amount of public money being diverted to private companies has not remained constant, as Dayan and Buckingham contend.^30^ Rather, Rowland calculates that “in total, between 2013/2014 and 2018/2019 an additional £5.6 billion of NHS England's budget went on the independent sector—an increase of 23%,” which was primarily due to an increase “in the amount that local Clinical Commissioning Groups (£4.3bn) have purchased from the independent sector.”^31^

The increase in private provision is important as, although it may benefit the coffers of private companies, studies indicate that it detrimentally affects the quality of health care.^32,33^ To use another metaphor from Ancient Greek literature, the Trojan horse (private companies) has already penetrated the gates of the city (the English NHS). According to Ancient Greek mythology, the inhabitants of Troy had an inkling of what would happen if the Mycenaean Greeks penetrated the walls of their city during the Trojan war, as the demi-god Herakles had reputedly already sacked that city–state previously.^34^ The work of Roderick and Pollock has already highlighted the problems with the increased involvement of the private sector within the English NHS, such as the negative impact on community services.^35^ Roderick and Pollock's latest analysis highlights how scholars can utilize the experience of managed care in the United States and elsewhere to learn the possible future for health care in England.^4^ As the UK government continues to valorize the founding principles of the NHS while enacting policies that undermine them, such principles are a means of critiquing government policy.^36^ As public experience increasingly diverges from such principles—which, as mentioned above, can be viewed as a moral economy pertaining to health care within England—there may be a crisis of legitimacy.^2^ As Roderick and Pollock state, the founding principles of the NHS also provide “a promising basis for continuing the vital and sustained campaigns to rebuild the NHS in England.”^4^ One can only hope that, unlike Cassandra, the warnings of Roderick and Pollock are heeded. The increase in campaigning activity against privatization of the English NHS indicates that such warnings are being heeded by many.^37^

## References

[1] Wellings D Jefferies D Maguire D, et al. Public Satisfaction with the NHS and Social Care in 2021: Results from the British Social Attitudes Survey. Kings Fund and the Nuffield Trust; 2022.

[2] Benbow D. The sociology of health and the NHS. Sociol Rev. 2017;65(2):416–422.

[3] Thompson E. The moral economy of the English crowd in the eighteenth century. Past Present. 1971;50(1):76–136.

[4] Roderick P Pollock A. Dismantling the NHS in England. Int J Health Serv. 2022;52(4):470–479. 10.1177/00207314221114540 PMC9449439 35876348

[5] Pollock A Leys C Price D Rowland D Gnani S. NHS PLC: The Privatisation of our Healthcare. Verso; 2005.

[6] Pollock A Price D Roderick P, et al. Health and social care bill 2011. Briefing 01a for House of Lords: clause 1. 2011.

[7] National Health Service H.C. Bill (2017–19) [250].

[8] Powell M. Who killed the English national health service. Int J Health Policy Manag. 2015;4(5):267–269. 25905477 10.15171/ijhpm.2015.72 PMC4417629

[9] Aesop. Aesop’s Fables. William Heineman; 1912.

[10] Homer. The Iliad. Penguin; 2003.

[11] Kotecha V. Dealing with the Legacy of PFI – Options for Policymakers. Centre for Health and the Public Interest; 2018.

[12] Centre for Health and the Public Interest. P.F.I. Profiting from Infirmaries. Centre for Health and the Public Interest; 2017.

[13] National Health Service (Procurement, Patient Choice and Competition) Regulations (No.2), SI 2013/500.

[14] Price C. From red to black: Private sector profiting as NHS crumbles. Pulse Today, October 17 2016.

[15] Thomas C, Poku-Amanfo V, Patel P. *The State of Health and Care 2022*. Institute for Public Policy Research; 2022.

[16] Health and Care Act 2022, S.18-S.25 and Sch.2 and Sch.3.

[17] Waitzkin H Hellander I. The history and future of neo-liberal health reform: Obamacare and its predecessors. Int J Health Serv. 2016;46(4):747–766. 27487835 10.1177/0020731416661645

[18] Himmelstein D Hellander I Woolhandler S. Bleeding the Patient: The Consequences of Corporate Healthcare. Common Courage Press; 2003.

[19] Iriart C Merhy E Waitzkin H. Managed care in Latin America: The new common sense in health policy reform. *Soc Sci Med*. 2001;52(8):1243–1253. 10.1016/s0277-9536(00)00243-4 11281407

[20] National Health Service Act 2006, S.14Z31(1) as amended by Health and Care Act 2022, S.20.

[21] Health and Care Act 2022, S.80(2).

[22] British Medical Association (BMA). The Role of Private Outsourcing in the Covid-19 Response. BMA; 2020.

[23] Health and Care Act 2022, sch.1B, paras 4 and 11(4)(b).

[24] Guy M. Demarketisation, deregulation, dejuridification: removing competition from the English NHS with the health and care bill. Lancaster University Law School Working Paper; 2021.

[25] Sanchez-Graells A. Are there any gains to be had from the proposed new provider selection model for NHS commissioning? August 23 2021. Available at: https://legalresearch.blogs.bris.ac.uk/2021/08/are-there-any-gains-to-be-had-from-the-proposed-new-provider-selection-model-for-nhs-commissioning/ Accessed July 6 2022.

[26] Muschell J. Health Economics Technical Briefing Note: Privatization in Health. World Health Organisation; 1995, p3.

[27] Woolhandler S Himmelstein D. Competition in a publicly funded healthcare system. Br Med J. 2007;335(7630):1126. 18048539 10.1136/bmj.39400.549502.94 PMC2099512

[28] Thompson J. Studies in the Theory of Ideology. University of California Press; 1984.

[29] Thompson J. Ideology and Modern Culture. Polity Press; 2007.

[30] Dayan M Buckingham H. Will the new health and social care bill privatise the NHS? July 15 2021. Available at: https://www.nuffieldtrust.org.uk/public/news-item/will-the-new-health-and-care-bill-privatise-the-nhs. Accessed July 6 2022.

[31] Rowland D. Flawed data? Why NHS spending on the independent sector may actually be much more than 7% October 1 2019. Available at: https://blogs.lse.ac.uk/politicsandpolicy/nhs-spending-on-the-independent-sector/. Accessed July 6 2022.

[32] GoodairBReevesA. Outsourcing healthcare services to the private sector and treatable mortality rates in England, 2013–20: An observational study of NHS privatisation. Lancet. 2022;7(7):E638–E646.10.1016/S2468-2667(22)00133-535779546

[33] Footman K, Garthwaite K, Bambra C, McKee M. Quality check: Does it matter for quality how you organize and pay for health care? A review of the international evidence. *Int J Health Serv*. 2014;44(3):479–505.

[34] Appollodorus. The Library. William Heineman; 1921.

[35] Pollock A. Interview. In McCartney M (ed) The State of Medicine: Keeping the Promise of the NHS. Pinter and Martin Limited; 2016: 120–122.

[36] Benbow D. An adornian ideology critique of neo-liberal reforms to the English NHS. J Political Ideol. 2021;26(1):59–80.

[37] Benbow D. Juridification, new constitutionalism and market reforms to the English NHS. Cap Cl. 2019;43(2):293–313.

